# Neural correlates of illness awareness in obesity: an exploratory pilot fMRI study

**DOI:** 10.3389/fneur.2026.1675116

**Published:** 2026-03-23

**Authors:** Jianmeng Song, Julia Kim, Danielle Bukovsky, Gianluca Volpe, Michaela Morra, Yasaman Kambari, Edgardo Carmona-Torres, Fumihiko Ueno, Caitlin Chow, Shannen Kyte, Sri Mahavir Agarwal, Margaret Hahn, Satya Dash, Sanjeev Sockalingam, David Urbach, Vincenzo De Luca, Bruce Pollock, Ariel Graff-Guerrero, Philip Gerretsen

**Affiliations:** 1 Multimodal Imaging Group, Brain Health Imaging Centre, Centre for Addiction and Mental Health (CAMH), Toronto, ON, Canada; 2 Institute of Medical Science, University of Toronto, Toronto, ON, Canada; 3 Schizophrenia Division, CAMH, Toronto, ON, Canada; 4 Department of Psychiatry, University of Toronto, Toronto, ON, Canada; 5 Banting and Best Diabetes Centre, University of Toronto, Toronto, ON, Canada; 6 Department of Pharmacology & Toxicology, University of Toronto, Toronto, ON, Canada; 7 Department of Clinical Medicine, Faculty of Health and Medical Sciences, University of Copenhagen, Copenhagen, Denmark; 8 Department of Medicine, University Health Network, Toronto, ON, Canada; 9 Department of Education, Centre for Addiction and Mental Health, Toronto, ON, Canada; 10 Department of Surgery, Temerty Faculty of Medicine, University of Toronto, Toronto, ON, Canada; 11 Department of Surgery, Women’s College Hospital, Toronto, ON, Canada; 12 Geriatric Psychiatry, CAMH, Toronto, ON, Canada

**Keywords:** blood oxygen level dependence, functional MRI, illness awareness, MRI, obesity, task-based MRI, weight Perception

## Abstract

**Introduction:**

Obesity is a complex condition that negatively impacts health, quality of life, and life expectancy. Nonadherence to existing clinical interventions remains a significant barrier for patients with obesity, with 80% of overweight individuals struggling to maintain long-term weight loss. Impaired illness awareness is a factor that may contribute to treatment nonadherence. Previous functional imaging studies in other conditions have suggested that impaired illness awareness may be related to altered activity or dysconnectivity in frontoparietal regions, including the posterior parietal area (PPA) and dorsolateral prefrontal cortex (dlPFC). As such, this exploratory study aimed to investigate the brain regions associated with impaired illness awareness in individuals with obesity.

**Methods:**

A total of 26 participants (Age = 50.4 (14.5), 75% female) with a mean BMI of 37.4 (SD = 5.1) were included. Participants completed an individually tailored subjective obesity awareness task during fMRI. The task paradigm consisted of a bank of brief stimuli, including ‘yes/agree’ or ‘no/disagree’ questions/statements derived from the core domains of illness awareness and control stimuli. Obesity awareness was assessed based on response accuracy to the paradigm’s obesity-related stimuli. Participants were also grouped into impaired (≤80% response accuracy, *n* = 14) versus intact obesity awareness (>80%, *n* = 12). Regression and non-parametric between-group analyses were conducted to assess brain activation, as measured by fMRI blood oxygen level dependent (BOLD) response during the obesity awareness task. Regions of interest for impaired obesity awareness were the PPA, dlPFC, and insula, controlling for age and gender.

**Results:**

Impaired subjective obesity awareness was related to increased BOLD responses in the left PPA, but not the dlPFC and insula during an obesity awareness fMRI task. Similarly, participants with impaired obesity awareness showed increased BOLD response to the obesity awareness task in PPA compared to those with intact obesity awareness.

**Discussion:**

Impaired obesity awareness may be related to increased brain activation in the PPA, though future replication is needed. Identifying neuroimaging biomarkers of impaired obesity awareness can help provide brain targets for intervention, with a goal of facilitating treatment adherence.

## 1 Introduction

Obesity, defined as a body mass index (BMI) ≥ 30 kg/m^2^, has been declared a global epidemic by the World Health Organization (WHO) (1). Obesity negatively impacts physical and mental health, reduces quality of life, and ranks as the fifth leading cause of mortality worldwide (2–4). Although effective obesity management interventions exist (i.e., dietary changes, increased physical activity), adherence to these interventions remains a significant barrier to positive clinical outcomes (5–10), with 80% of overweight individuals struggling to maintain long-term weight loss (11). Various social, environmental, and behavioral factors affect the prevalence of obesity, and an individual’s willingness to seek and adhere to treatment (12, 13).

Larger body sizes are increasingly normalized (14) as obesity rates continue to rise, with nearly three-quarters of US adults classified as overweight or obese (15). This shift in social norms appears to contribute to the growing trend of weight misperception, where many adults with obesity underestimate their weight, and do not identify themselves as having obesity (16–20). While the contemporary body acceptance, ‘body-positivity’ movement has helped reduce weight related stigma (21, 22), the reinforcement of these norms may unintentionally contribute to negative health consequences, such as increasing the incidence of obesity and impaired obesity awareness. That is, with the normalization of obesity, individuals within this weight categorization will be less likely to perceive their weight as a health concern (23). Individuals who fail to recognize that they have obesity may be described as having anosognosia or impaired illness awareness (24, 25). Impaired illness awareness involves several core components, including impaired general obesity awareness (e.g., “I am overweight”), symptom awareness and accurate attribution to the illness (e.g., “my back pain is related to my weight”), awareness of the need for treatment (e.g., “I would benefit from exercise”), and awareness of the negative consequences of obesity (e.g., “my weight may lead to heart disease”) (26–29). Impaired illness awareness reflects a subjective denial of obesity as a medical condition that persists even after individuals receive education about the condition (29). This impairment is associated with negative or indifferent attitudes toward weight loss and a resistance to lifestyle or behavioral interventions (6, 30–36). Of note, individuals who are overweight or obese and underestimate their weight are 65 to 85% less likely to want to lose weight compared to those with an accurate weight perception (23, 32).

Although impaired subjective obesity awareness is a notable barrier to effective obesity treatment, its neurobiological basis remains an understudied and poorly articulated concept. Growing evidence suggests that impaired illness awareness may be related to dysfunction in frontoparietal brain regions involved in self-referential processing and cognitive control. Supporting this, impaired illness awareness has been reported after lesions in frontoparietal and insular areas secondary to stroke, traumatic brain injury, and neurodegenerative disorders (24, 25, 37–44). Beyond conditions attributable to structural lesions, schizophrenia provides a well-studied ‘functional’ clinical model for understanding the neural basis of impaired illness awareness, particularly through functional neuroimaging. Specifically, impaired illness awareness in schizophrenia is associated with altered activity and connectivity in the frontoparietal and insular regions, as measured with functional magnetic resonance imaging (fMRI) (40, 45–48).

To our knowledge, no studies to date have explored the neural correlates of impaired obesity awareness. In this exploratory pilot fMRI study, we aimed to investigate the brain areas related to impaired obesity awareness as assessed by an illness awareness fMRI task during scanning. We hypothesized that, similar to previous findings in other conditions, impaired obesity awareness would be associated with activation in frontoparietal and insular regions, as measured by the blood-oxygen-level-dependent (BOLD) response to the illness-awareness task.

## 2 Methods

### 2.1 Participants

Adult participants with BMI ≥ 30 kg/m^2^ and scores ≥32 on the Wide Range Achievement Test-III (WRAT III) (49) Word Reading subtest were recruited through the Centre for Addiction and Mental Health (CAMH), University Health Network, and community via Facebook and Kijiji advertisements. WRAT-III is used as an index of reading ability and pre-morbid IQ (39, 50–52). The cut off score was selected to ensure participants had the minimum level of reading literacy to understand the study assessments and fMRI paradigm (50). Participants provided written informed consent to participate in the study in accordance with the guidelines set out by the Research Ethics Board of CAMH.

Exclusion criteria were: unable to fit in the MRI scanner; contraindications to MRI; any concomitant major medical illness or neurological illness, or active psychiatric illness based on the Mini-International Neuropsychiatric Interview (MINI) (53); or positive urine drug test.

### 2.2 Study measures

Subjective obesity awareness was measured using the Obesity Awareness and Insight Scale (OASIS) (29), a self-report scale that has been developed and psychometrically tested to measure the four core dimensions of illness awareness: general illness awareness, symptom attribution, awareness of need for treatment, awareness of negative consequences.^1^ To address prior knowledge and education about obesity, prior to the assessment of subjective obesity awareness (i.e., administration of OASIS), participants were provided with basic education on obesity, including its definition, symptoms, health risks, and evidenced-based intervention strategies. This was done to ensure the assessment of subjective obesity awareness and level of acceptance of the condition, and not participant’s degree of knowledge about the disease. The educational material is available at the above web address.

Illness awareness was assessed based on fMRI paradigm accuracy, which was calculated as the proportion of correct responses to the illness awareness task stimuli (See Section 2.3.2 Illness awareness paradigm). Those with impaired obesity awareness was defined as having < 80% accuracy for illness-related stimuli (i.e., without control stimuli), while those with intact illness awareness were defined as having illness-related stimuli accuracy ≥ 80%. As the task-based measure captures behavioral performance on the same stimuli presented during scanning, it offered a direct and temporal index of the brain activation under investigation and therefore was selected to be used as the independent variable in the proposed analyses.

Illness perception was also measured with the Brief Illness Perception Questionnaire (Brief IPQ) (54), which is a shorter version of the revised Illness Perception Questionnaire. The Brief IPQ measures different aspects of illness perception, indirectly covering four of the core domains of illness awareness, i.e., consequences, timeline, personal control, treatment control, identity concern, understanding, and emotional responses. Other clinical assessments included the Beck Depression Inventory (BDI) (55), Generalized Anxiety Disorder 7-item scale (GAD-7) (56), and Binge Eating Scale (BES) (57). Demographic information such as age, gender, and BMI was also collected.

### 2.3 MRI data acquisition

The MRI was performed with a 3 T GE scanner (General Electric, Waukesha, WI) equipped with standard 8-channel head coils and sequences at CAMH. Head motion was minimized by mounting foam padding equally around the head-coil. For localization purposes, a high-resolution structural T1-weighted IR-Prepped 3D FEDR anatomical images (120 contiguous axial 1.1-mm-thick slices) were acquired (BRAVO, TR = 6.7 ms; TE = 3 ms; flip-angle 8°; 256 × 256 matrix; FOV = 23 cm; slice thickness = 0.9 mm) to assess brain structure.

#### 2.3.1 Functional imaging

fMRI scans consisted of BOLD-sensitive pulse sequence with whole-brain coverage. In the functional imaging session, 215 volumes (37 contiguous axial 4.0-mm-thick slices) covering the whole brain were acquired using a T2*-sensitive echo planar imaging (EPI) sequence (TR = 2,300 ms; TE = 30 ms; flip-angle 60°; 64 × 64 matrix; FOV = 20 cm). Fieldmaps were obtained using a dual-echo gradient-echo sequence (50 contiguous axial 4.0-mm-thick slices; TE₁ = 6.5 ms, TE₂ = 8.5 ms; TR = 1,000 ms; flip angle 60°; 64 × 64 matrix; FOV = 20 cm). The resulting phase-difference maps were used to compute voxel displacement maps for distortion correction during preprocessing.

#### 2.3.2 Illness awareness paradigm

**Figure 1:**
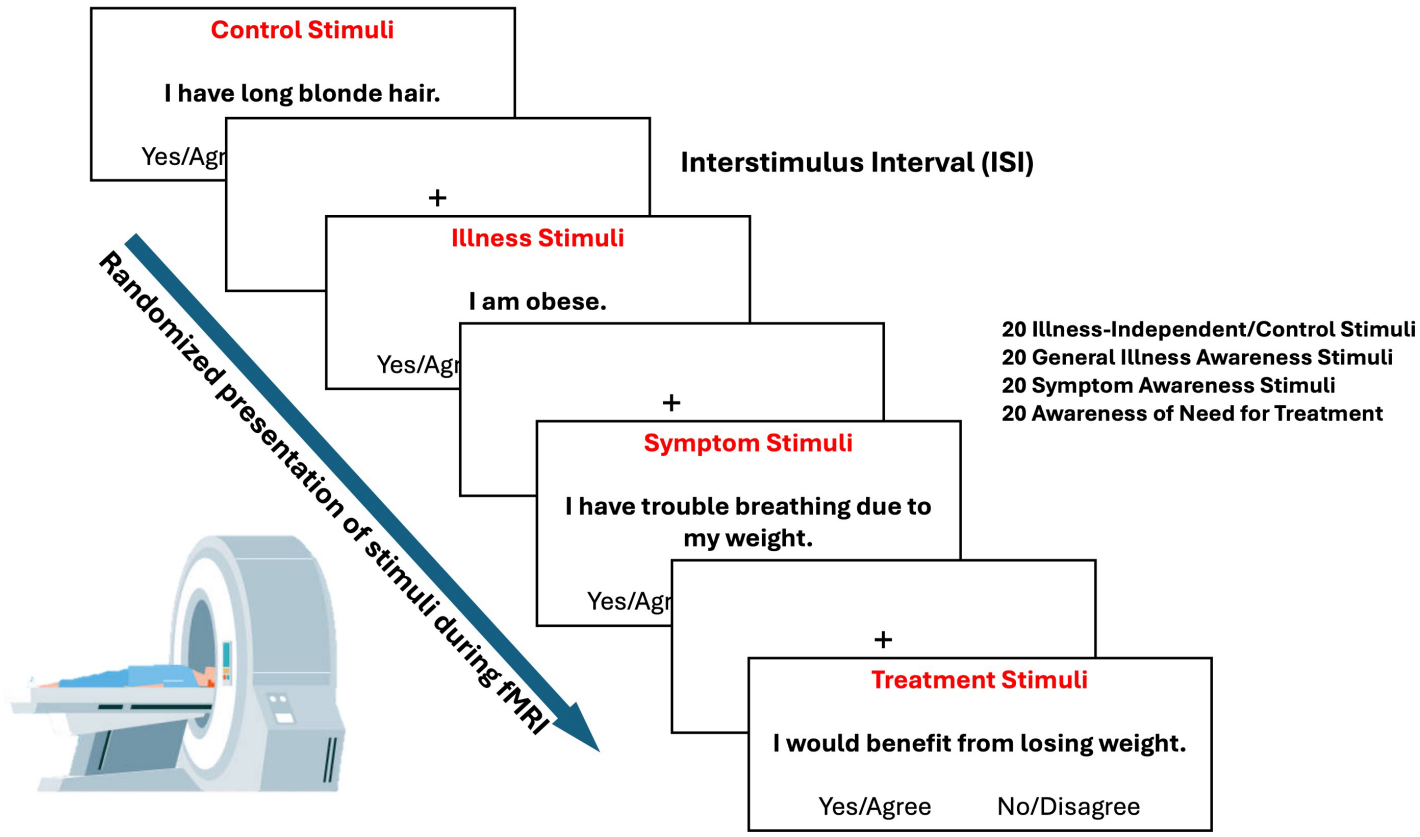
Functional MRI task designed to confront participants with their beliefs about their illness. An adjustable mirror located above the participant’s eyes was used to view the statements projected onto a screen placed at the head of the bed using E-Prime software (Psychology Software Tools, Pittsburgh, PA). Each statement was presented for 4 s, with a variable interstimulus interval of 2 s on average with a fixation cross. Each participant was outfitted with an MR-compatible button-box.

During the MRI scan, participants underwent a tailored fMRI paradigm designed to confront participants with their beliefs about their obesity (Figure 1). A similar paradigm was employed in prior work from our group to explore the functional neuroanatomy of impaired illness awareness in other conditions (39, 51, 52). The paradigm consisted of a bank of 80 ‘yes/agree’, ‘no/disagree’ questions/statements derived from four categories: general illness awareness (20 items), obesity symptom attribution (20 items), awareness of the need for treatment (20 items), and independent/control stimuli (20 items). About 75% of the paradigm (i.e., control, general illness awareness, and need for treatment awareness items) were common across all participants, whereas the remaining items (25%) were symptom-specific that were intentionally tailored to the content of the participants’ own experiences or symptoms associated with obesity identified from the OASIS. The symptom specific items are structurally the same between participants, but distinct in terms of the obesity-related experiences.

The paradigm was designed to isolate brain activities related with illness awareness-related processing by contrasting the BOLD responses between illness awareness-related stimuli (requiring self-reflection on obesity-related beliefs, awareness, and appraisal) and control stimuli that were identical in structure and process (i.e., required self-reflection), but did not engage illness awareness.

Before scanning, participants practiced responding to sample questions on a laptop using E-prime software (Psychology Software Tools, Pittsburgh, PA) to ensure that they understood the procedure and to confirm the symptom-specific content reflected their experiences. During the scan, each participant was outfitted with an MR-compatible button-box for pressing ‘yes/agree’, ‘no/disagree’ with right index and middle fingers, respectively. An adjustable mirror located above the participant’s eyes was used to view the stimuli projected onto a screen placed at the head of the bed. Stimuli were presented on the monitor inside the MRI scanner using E-Prime. Each statement was presented for 4 s, with a variable interstimulus interval of 2 s on average with a fixation cross. Participants could respond up to 5 s following the presentation of the stimulus. Participants were instructed to answer intuitively in a timely manner.

### 2.4 Image preprocessing

#### 2.4.1 Functional images

The data were preprocessed and analyzed using Statistical Parametric Mapping 12 tool (SPM12) (58). The first three functional image slices collected during the task initiation countdown period were discarded to allow for T1 equilibrium effects. Data from the remaining 212 volumes were used in the analysis. The functional images were first slice-timing adjusted with the 2nd slice as the reference, then realigned with the preprocessed fieldmaps using the SPM12 Fieldmap toolbox. The generated mean realigned images were coregistered with their corresponding T1 structural images segmented from 6 tissue maps (SPM12 templates) and eventually normalized into the standard stereostatic space with the Montreal Neurological Institute (MNI) EPI template. Computed transformation parameters were applied to all functional images with 3 mm^3^ isotropic voxel interpolation. The resulting images were smoothed using an 8-mm full-width half-maximum isotropic Gaussian kernel.

#### 2.4.2 First-level analyses

Similar to the analyses performed in other studies in our group investigating the neural correlates of illness awareness (53), the first-level contrasts were created using random-effect analyses between all illness awareness-related stimuli (i.e., general illness awareness, symptom attribution, and awareness of the need for treatment combined) and illness-independent/control stimuli, as well as between each illness awareness subcategory and illness-independent/control stimuli. These contrast images were then used for second-level whole brain analyses.

### 2.5 Whole brain analyses

For second-level analyses, regression analyses were conducted as the primary analysis to investigate the neural correlates of impaired obesity awareness (i.e., paradigm illness-related stimuli accuracy as a continuous variable). Two sample *t*-tests were also performed exploratorily as a sensitivity analysis to investigate differences in BOLD-response between intact (paradigm illness-related stimuli accuracy ≥ 80%) and impaired illness awareness groups (paradigm illness-related stimuli accuracy < 80%). All models included age and gender as covariates.

As the *a priori* hypothesis was specific to the posterior parietal area (PPA) (39, 51, 59), dorsolateral prefrontal cortex (dlPFC) (51), and insula (37, 38, 48), we confined the statistical search to these regions of interest (ROIs) using masks (60) from the Automated anatomical labelling (AAL) atlas 2 (61): bilateral angular gyri, inferior parietal regions, supramarginal gyri, superior parietal regions, and insulae. Another six *a priori* ROIs were chosen in these regions based on findings from our prior neuroimaging studies by confining a 10 mm sphere around identified peaks: bilateral angular gyri [(±46, −70, 36) (40), (±42, −80, 30) (39), (±44, −60, 40) (51)], inferior parietal regions (±36, −64, 42) (39), and dlPFC [( ± 27, 49, 24) (51), (±14, 34, 42) (39)] using the Talairach Daemon atlas with WFU-Pickatlas software (84–86).

The threshold was set at *p* ≤ 0.001 level of significance (*t* > 3.69), 0 voxels. A cluster was reported as significant if the peak survived a familywise error (FWE) small volume correction for multiple comparisons of *p* ≤ 0.05 (61) within *a priori* ROIs. For exploratory purposes, the coordinates for any peaks outside of our ROIs for impaired illness awareness and intact illness awareness were reported. The locations of these peaks were labelled based on the AAL atlas.

### 2.6 Statistical analysis

Statistical analyses of clinical, demographic, and behavioral variables were carried out with SPSS software (version 29.0). Group comparisons using independent sample *t*-test were conducted between the impaired and intact illness awareness groups. Bivariate Pearson correlations were performed between illness awareness (OASIS) scores and paradigm illness-related stimuli accuracy.

## 3 Results

### 3.1 Demographical and clinical data

Data for four participants could not be used due to neuroimaging data loss (*n* = 2), possible diagnosis of schizophrenia (*n* = 1), and excessive head motion (*n* = 1). The final sample consisted of 26 participants (mean age = 49.2, SD = 14.3, range: 19–71) with 14 in the impaired illness awareness and 12 in the intact illness awareness groups. The demographic and clinical data, and group comparisons are presented in Table 1. Independent sample *t*-tests found that the impaired obesity awareness group scored significantly lower than the intact obesity awareness group on the OASIS average (*t* = −3.25, *p* = 0.004), symptom attribution (*t* = −2.61, *p* = 0.018), and awareness of need for treatment subscale (*t* = −2.89. *p* = 0.008), and Brief IPQ scores (*t* = −2.12, *p* = 0.045). Significant differences in Brief IPQ scores observed between the impaired and intact obesity awareness groups supports the convergent validity of participants’ paradigm performance. The two groups did not differ in other demographic characteristics, BMI or clinical measures. Pearson correlation between OASIS average score and illness-related paradigm accuracy was *r* = 0.65 (*p* = 0.001).

**Table 1:** Demographic and clinical characteristics.

	Mean (SD) or %		Group comparison^b^	
	Impaired^a^	Intact^a^	*t* value	*p* value
*n*	14	12	–	–
Age	52.1 (15.2)	45.8 (12.9)	1.13	0.269
Gender (%female)	85.7	66.7	-	0.365^c^
Education (years)	15.9 (2.4)	15.4 (1.2)	0.58	0.570
Smoking status (%smoker)	0	8.3	–	–
Ethnicity (%)				
Caucasian	85.7	58.3	–	–
African descent	0	8.3	–	–
East Asian	0	8.3	–	–
Hispanic	0	8.3	–	–
Other	14.3	16.7	–	–
BMI	36.3 (4.9)	38.6 (5.5)	−1.11	0.280
GAD-7	2.8 (2.4)	2.7 (3.7)	0.10	0.921
BDI	4.4 (4.8)	6.1 (6.0)	−0.81	0.424
Brief IPQ	35.4 (8.8)	43.6 (10.6)	−2.12	0.045*
BES	9.3 (6.8)	11.5 (8.6)	−0.73	0.471
OASIS average score	6.1 (2.1)	8.2 (1.0)	−3.25	0.004*
Illness awareness subscale	7.3 (2.2)	8.8 (1.3)	−1.96	0.063
Symptom attribution subscale	4.5 (3.6)	8.1 (1.1)	−2.61	0.018*
Need for treatment subscale	5.8 (2.1)	8.0 (1.5)	−2.89	0.008*
Negative consequences subscale	6.5 (3.1)	8.1 (2.7)	−1.36	0.188
fMRI paradigm response accuracy (%)	73.2 (7.1)	86.5 (4.0)	−6.14	<0.001*
All illness-related stimuli	67.5 (9.4)	85.2 (4.0)	−6.39	<0.001*
Illness awareness stimuli	92.1 (7.5)	95.0 (8.0)	−0.94	0.357
Symptom attribution stimuli	31.2 (20.4)	71.1 (11.3)	−6.28	<0.001*
Need for treatment stimuli	79.1 (12.2)	89.5 (7.5)	−2.67	0.014*
Control stimuli	90.4 (6.0)	90.4 (7.8)	−0.02	0.983
fMRI paradigm reaction time (sec)				
Total illness-related stimuli	2.6 (5.4)	2.4 (0.6)	1.13	0.270
Illness awareness stimuli	2.2 (0.7)	1.9 (0.6)	1.24	0.227
Symptom attribution stimuli	3.1 (0.6)	2.9 (0.6)	0.79	0.437
Need for treatment stimuli	2.6 (0.5)	2.4 (0.6)	1.16	0.259
Control stimuli	1.9 (0.5)	1.8 (0.5)	0.97	0.343

a Impaired obesity awareness group: obesity-related paradigm (i.e., without control stimuli) accuracy <80%; Intact obesity awareness group: paradigm accuracy ≥80%.

b Independent sample *t*-test.

c Fisher’s exact test for gender as the categorical variable.

BMI: Body mass index; GAD-7: Generalized anxiety disorder 7-item scale; BDI: Beck depression inventory; Brief IPQ: Brief illness perception questionnaire; BES: Binge eating scale; OASIS: Obesity awareness and insight scale. *p < 0.05.

### 3.2 Functional imaging results

#### 3.2.1 Whole brain analyses

The results for the regression and group analyses are presented in Tables 2, 3, and Supplementary Tables 1, 2.

**Table 2:** Regional activations for the second-level contrasts—regression.

Impaired	Adjusted for age, gender							
	Cluster Maxima			Cluster size	*t* value	*η_p_*^2^ [95%CI] (df = 22)	*p* value uncorrected	*p* value FWE small volume corrected
	*x*	*y*	*z*					
Illness-related > Control								
Left angular gyrus	−42	−58	23	18	4.81	0.51 [0.29, 0.69]	<0.001	0.007*
Right angular gyrus	54	−64	23	4	4.29	0.46 [0.24, 0.65]	<0.001	0.027*
Right supramarginal gyrus	54	−37	23	3	3.89	0.41 [0.20, 0.61]	<0.001	0.063
General illness awareness > Control								
Left angular gyrus	−39	−58	23	40	6.84	0.68 [0.40, 0.85]	<0.001	<0.001*
Right angular gyrus	39	−67	44	12	4.20	0.45 [0.13, 0.71]	<0.001	0.031*
Right supramarginal gyrus	66	−22	17	2	3.63	0.37 [0.08, 0.66]	0.001	0.098
Left superior frontal region	−12	17	56	1	3.57	0.37 [0.07, 0.66]	0.001	0.206
Right superior frontal region	21	32	53	1	3.76	0.39 [0.09, 0.67]	0.001	0.159
Left middle frontal region	−33	17	29	1	3.92	0.41 [0.10, 0.69]	<0.001	0.124
Right insula	39	−22	2	1	3.59	0.37 [0.07, 0.66]	0.001	0.108
(Gerretsen et al., 2014)								
Left angular gyrus (−46, −70, 36)	−42	−64	29	2	3.80	0.40 [0.19, 0.60]	<0.001	0.021*
Right angular gyrus (46, −70, 36)	39	−70	44	6	4.12	0.44 [0.22, 0.64]	<0.001	0.012*
(Gerretsen et al., 2015)								
Left inferior parietal region (−36, −64, 42)	−36	−64	32	1	3.75	0.39 [0.09, 0.67]	0.001	0.024*
Right inferior parietal region (36, −64, 42)	39	−67	44	8	4.20	0.45 [0.13, 0.71]	<0.001	0.010*
(Kim et al., 2019)								
Left angular gyrus (−44, −60, 40)	−45	−61	29	1	3.98	0.42 [0.11, 0.69]	<0.001	0.015*
Right angular gyrus (44, −60, 40)	39	−67	44	9	4.20	0.45 [0.13, 0.71]	<0.001	0.010*
Symptom attribution > Control								
Left angular gyrus	−39	−58	23	9	4.21	0.45 [0.13, 0.71]	<0.001	0.023*
Right angular gyrus	54	−64	23	3	4.01	0.42 [0.11, 0.70]	<0.001	0.048*
Left inferior parietal region	−45	−28	41	3	3.73	0.39 [0.09, 0.67]	0.001	0.105
Left insula	−30	−28	20	1	3.55	0.36 [0.07, 0.66]	0.001	0.128
Right insula	33	23	14	1	3.57	0.37 [0.07, 0.66]	0.001	0.121
Need for treatment > Control								
Left angular gyrus	−39	−58	23	11	4.83	0.51 [0.19, 0.75]	<0.001	0.006*
Right angular gyrus	51	−61	23	3	3.72	0.39 [0.09, 0.67]	0.001	0.077

Threshold: *p* < 0.001, uncorr., 0 voxels. *η_p_*^2^: Partial *η*^2^. CI: confidence interval. All regions in association with impaired obesity awareness were corrected for using a priori ROIs based on the AAL atlas and peaks identified in prior studies. No suprathreshold peaks for the contrast intact > impaired obesity awareness were identified. *p < 0.05.

**Table 3:** Regional activations for the second-level contrast—group comparison.

Adjusted for age, gender								
Impaired > Intact	Cluster Maxima			Cluster size	*t* value	*η_p_*^2^ [95%CI] (df = 22)	*p* value uncorrected	*p* value FWE small volume corrected
	*x*	*y*	*z*					
Illness-related > Control								
Left angular gyrus	−45	−61	23	11	4.37	0.46 [0.15, 0.72]	<0.001	0.017*
Left inferior parietal region	−36	−46	50	1	3.53	0.36 [0.07, 0.66]	0.001	0.148
Right supramarginal gyrus	60	−40	23	3	3.81	0.39 [0.09, 0.67]	<0.001	0.078
General Illness awareness > Control								
Left angular gyrus	−42	−58	23	15	4.92	0.52 [0.20, 0.76]	<0.001	0.006*
Right angular gyrus	39	−70	44	2	4.06	0.43 [0.12, 0.70]	<0.001	0.044*
(Gerretsen et al., 2014)								
Right angular gyrus (46, −70, 36)	39	−70	44	2	4.06	0.43 [0.12, 0.70]	<0.001	0.014*
(Gerretsen et al., 2015)								
Right medial superior frontal gyrus (14, 34, 42)	18	35	50	1	3.51	0.36 [0.07, 0.66]	<0.001	0.039*
Right inferior parietal region (36, −64, 42)	39	−70	44	2	4.06	0.43 [0.12, 0.70]	<0.001	0.014*
(Kim et al., 2019)								
Right angular gyrus (44, −60, 40)	43	−67	44	1	3.68	0.38 [0.08, 0.67]	<0.001	0.028*
Symptom attribution > Control								
Left angular gyrus	−48	−61	23	4	3.88	0.40 [0.09, 0.68]	<0.001	0.045*
Right supramarginal gyrus	60	−40	23	2	3.54	0.36 [0.07, 0.66]	0.001	0.126
Left superior parietal region	−27	−52	62	2	3.72	0.39 [0.09, 0.67]	0.001	0.096
(Gerretsen et al., 2015)								
Right angular gyrus (42, −80, 30)	36	−76	32	1	3.52	0.36 [0.07, 0.66]	<0.001	0.037*
Need for treatment > Control								
Left angular gyrus	−45	−61	23	5	4.14	0.44 [0.13, 0.70]	<0.001	0.026*
Right superior parietal region	18	−61	62	1	3.72	0.39 [0.09, 0.67]	0.001	0.098
Right insula	36	20	−13	3	3.85	0.40 [0.09, 0.68]	<0.001	0.072

Threshold: *p* < 0.001, uncorr., 0 voxels. *η_p_*^2^: Partial *η*^2^. CI: confidence interval. All regions in association with impaired obesity awareness were corrected for using a priori ROIs based on the AAL atlas and peaks identified in prior studies. No suprathreshold peaks for the contrast intact > impaired obesity awareness were identified. *p < 0.05.

##### 3.2.1.1 Regression analysis

The results for the contrast total illness awareness (general illness + symptom + need for treatment) > control revealed significant peak BOLD responses in left and right angular gyri (left: *t* = 4.81, *p* = 0.007; right: *t* = 4.29, *p* = 0.027; FWE corr.) associated with impaired obesity awareness (Table 2). Peak BOLD responses in the right supramarginal gyrus were also observed, which did not survive FWE correction.

**Figure 2:**
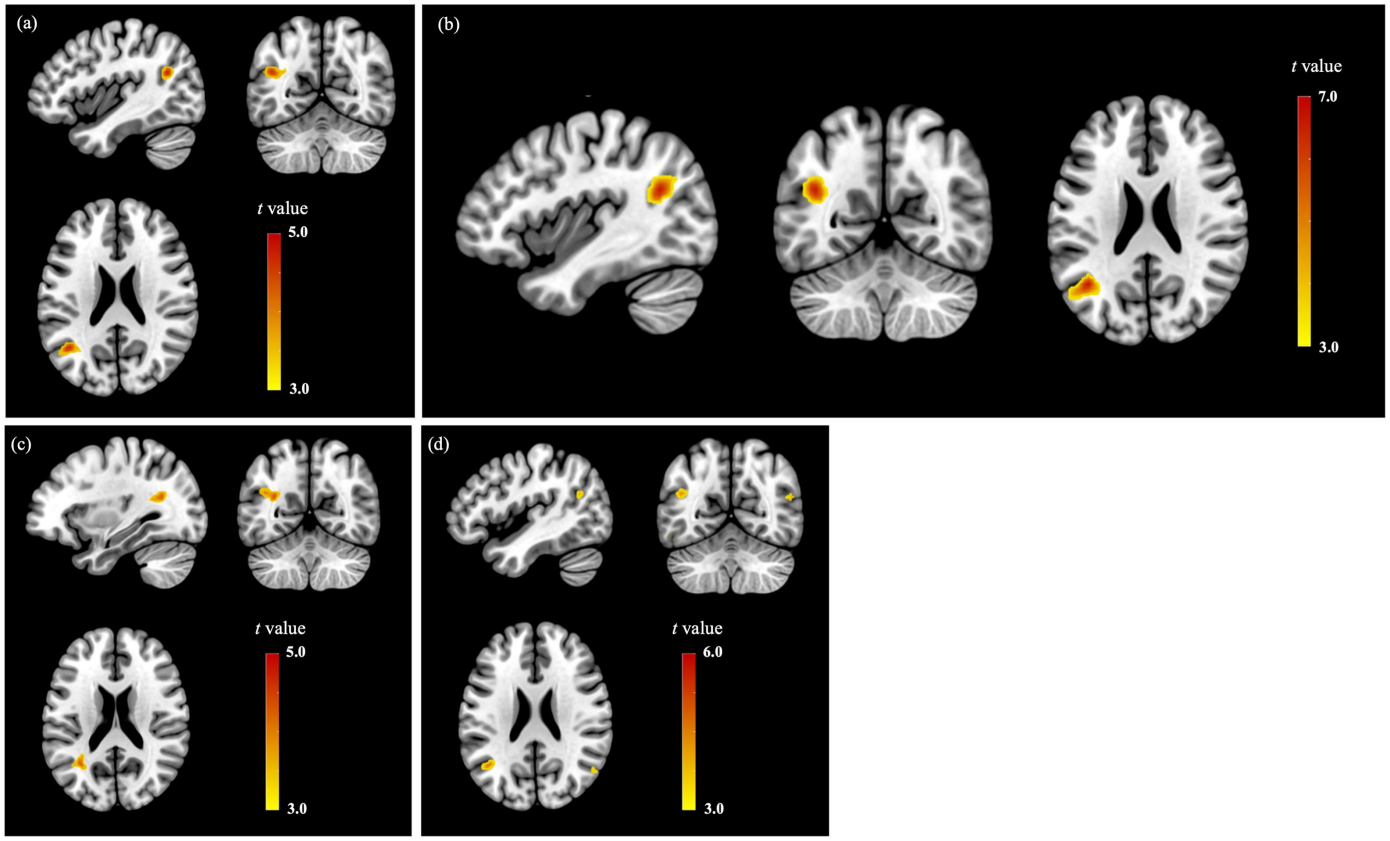
Brain activation in association with impaired obesity awareness for the contrasts: **(A)** Total illness awareness (general illness + symptom + need for treatment) > control stimuli (*p* < 0.001, voxel size ≥40); **(B)** General illness awareness > control stimuli (*p* < 0.001, voxel size ≥ 50); **(C)** Symptom awareness > control stimuli (*p* < 0.001, voxel size ≥ 50); **(D)** Awareness of need for treatment > control stimuli (*p* < 0.001, voxel size > 0). A low threshold of *p* < 0.001 was used to present all associated regions. Peaks are revealed in the left angular gyrus (-39, -58, 23).

The results of the contrasts general illness awareness > control, symptom attribution > control, and awareness of need for treatment > control revealed similar peak brain activations in the angular gyri associated with impaired obesity awareness (Table 2, Figure 1). Specifically, activations within the left and right angular gyrus were significant for both of the general illness awareness > control (left: *t* = 6.84, *p* < 0.001; right: t = 4.20, *p* = 0.031; FWE corr.) and symptom attribution > control contrasts (left: *t* = 4.21, *p* = 0.023; right: *t* = 4.01, *p* = 0.048; FWE corr.), while only activation within the left angular gyrus was significant for the awareness of need for treatment contrast (*t* = 4.83, *p* = 0.006; FWE corr.). Additionally, significant peak BOLD responses emerged for the contrast general illness awareness > control in relation to impaired obesity awareness in the following *a priori* ROIs based on MNI coordinates from our prior studies: bilateral angular gyri (±46, −70, 36 (38); ±44, −60, 40 (53)) and bilateral inferior parietal regions (±36, −64, 42) (37). Although other peak BOLD responses were observed for the obesity awareness subdomain contrasts in relation to impaired obesity awareness, none survived FWE correction: (i) activation in the right supramarginal gyrus, bilateral superior frontal regions, left middle frontal region and right insula for the contrast general illness awareness > control; (ii) activation in the left inferior parietal region and bilateral insulae for the contrast symptom attribution > control; (iii) activation in the right angular gyrus for the contrast awareness of need for treatment > control (Figure 2).

Exploratory analyses of brain activation associated with impaired and intact illness awareness in regions outside of *a priori* ROIs are presented in Supplementary Table 1. Although several peak activations other than the above mentioned were identified, none survived FWE correction.

##### 3.2.1.2 Group comparison

The results for the comparison between participants with impaired versus intact illness awareness (i.e., impaired illness awareness > intact illness awareness) for the same contrast used in the regression analysis, i.e., total illness awareness (general illness + symptom + need for treatment) > control revealed a positive association between BOLD response in the left angular gyrus and impaired obesity awareness (*t* = 4.37, *p* = 0.017, FWE Corr., Table 3). Additional associations between brain activation in left inferior parietal region and right supramarginal gyrus and impaired illness awareness were also identified, although these did not survive FWE correction (Table 3).

For the subdomain contrasts (general illness awareness > control, symptom attribution > control and awareness of need for treatment > control), the group comparison between participants with impaired versus intact illness awareness revealed BOLD-responses consistent with the findings in the regression analysis (Table 3). Notably, the following brain activations were also observed: (i) activation in right angular gyrus for the contrast general illness awareness > control (*t* = 4.06, *p* = 0.044, FWE corr.) and significant activation in the following *a priori* ROIs based on MNI coordinates from our prior studies: right angular gyrus (±46, −70, 36 (40); ±44, −60, 40 (51)), right inferior parietal region (36, −64, 42) (39), and right medial superior frontal gyrus (14, 34, 42) (39); (ii) Significant activation in right angular gyrus based on a priori ROI (42, −80, 30) (39), as well as activation that did not survive FWE correction in right supramarginal gyrus and left superior parietal region for the contrast symptom attribution > control; and (iii) activation in right superior parietal region and right insula for the contrast awareness of need for treatment > control, which also did not survive FWE correction.

Exploratory analyses of brain activation in regions outside of *a priori* ROIs are reported for all contrasts in Supplementary Table 2. No suprathreshold clusters for the comparison between participants with intact illness awareness versus IIA (i.e., intact illness awareness > impaired illness awareness) was identified.

## 4 Discussion

Impaired subjective obesity awareness impedes optimal treatment engagement and successful clinical outcomes (16, 20, 62, 63). Obese individuals with impaired illness awareness are less likely to recognize the severity of their illness and adhere to weight loss interventions (6, 31, 32, 34–36). This exploratory pilot study aimed to investigate the neural correlates of subjective obesity awareness in individuals with obesity during an fMRI illness awareness task. Our results show that individuals with impaired obesity awareness exhibited increased brain activation (i.e., increased BOLD responses) within the PPA, particularly the bilateral angular gyri. The PPA has been recognized as part of the default mode network (DMN), a network of brain regions active at rest in the absence of task-related stimuli, and which is suggested to be involved in self-referential processing (64, 65). On the other hand, although frontal involvement has been consistently reported in the prior literature, we did not observe significant BOLD responses in the *a priori* dlPFC regions that survived FWE correction. This may be attributable, in part, to the modest sample size, which may have limited statistical power to detect more subtle effects. Alternatively, it is possible that frontal engagement was less prominent in obesity-related awareness processing in the context of the present fMRI task. In addition, the absence of significant frontal findings may be related to the study design and analysis of our obesity awareness fMRI task (i.e., contrasting illness-related > control stimuli). While the prefrontal cortex is broadly implicated in domain-general processes such as working memory and cognitive control (66–68), our task was explicitly designed to isolate illness awareness–related neural activity while minimizing demands on these cognitive control processes. As a result, frontal contributions associated with domain-general cognitive processes may have been attenuated by the paradigm.

To our knowledge, this is the first exploratory study of its kind to assess neuroimaging correlates of subjective obesity awareness using an obesity awareness specific fMRI task. The results of this study align with the impaired illness awareness literature in other disorders, such as stroke, neurodegenerative disorders, and schizophrenia, which similarly indicate the involvement of frontoparietal regions in impaired illness awareness (41, 43, 44, 69, 70). For instance, two prior studies by our group that investigated the neural correlates of impaired schizophrenia awareness using a similar fMRI task had shown increased BOLD response in the left PPA (39, 51) and medial prefrontal cortex in relation to impaired illness awareness (39). Another study by our group revealed impaired illness awareness was associated with increased resting-state functional connectivity in the DMN with the left angular gyrus (i.e., left PPA) and in the self-referential network with the left insula (59). In stroke, frontoparietal and insular lesions are commonly associated with IIA (41, 43, 44, 71, 72).

Collectively, the findings suggest that impaired illness awareness across distinct conditions may stem from similar frontoparietal network dysfunction associated with the DMN and self-referential networks (40, 73, 74), though further replication is needed in other samples that have obesity and other conditions that feature impaired illness awareness. In schizophrenia, overactivity, or reduced suppression of the BOLD signal, in the DMN during self-reflection tasks has been linked to impaired self-awareness or excessive self-referential processing (40, 73). Extending this line of evidence, the present study found increased BOLD response in DMN-related frontoparietal regions during an obesity awareness task, suggesting the neural networks underlying impaired illness awareness may be generalizable to other conditions in which impaired problem or illness recognition may occur.

Frontoparietal brain regions, particularly the left PPA (with further investigation and replication of the results), may represent targets for intervention in randomized controlled experiments to establish brain-behavior causality. Improving impaired obesity awareness may facilitate adherence to weight management strategies. For instance, there has been increased interest in employing non-invasive brain stimulation, such as transcranial direct current stimulation (tDCS) and repetitive transcranial magnetic stimulation, to improve impaired illness awareness in various conditions. Meta-analytic evidence from single- and multi-session studies, as well as case reports, has demonstrated the feasibility of using tDCS to improve impaired illness awareness in other conditions, such as schizophrenia, by targeting frontoparietal regions (75–78). While the results of the current study are preliminary, if replicated in larger samples, they could represent neuroimaging biomarkers and therapeutic targets to improve subjective obesity awareness.

There are several limitations of the present study. First, our small sample size limited the statistical power to detect significant findings and to conduct multi-level statistical analyses. Additionally, our study is not designed to provide reliable estimates of effect size (79). It has been suggested that effect sizes are best assessed by applying predictive models—such as machine learning or multivoxel pattern analysis—to independent samples using cross-validation methods (79). Future studies with larger cohorts will be necessary to support such approaches and to evaluate the effect size of the findings. Second, given the nature of the study population, participants with obesity may experience discomfort in the MRI scanner due to body size, which could affect their ability to remain still and fully engage with the task, potentially influencing task performance. Nonetheless, only one participant’s data was unusable due to head motion. Future studies with larger sample sizes can apply stricter motion censoring criteria. Third, there are other factors that may be related to impaired obesity awareness, such as cognitive function, or cultural background (80) and many rudimentary and complex behaviors that engage the frontoparietal network, such as binge eating behavior (81, 82). That being said, the paradigm used in the current study was designed to isolate brain activation related to illness awareness-related processing. Future studies with larger and more diverse samples are warranted to account for these potential covariates. Fourth, the 80% accuracy threshold used for group comparisons was not based on a clinically validated cutoff of obesity awareness and should be interpreted with caution. This division was selected to facilitate complementary descriptive analysis and was not used as the basis for primary inference. Future research is needed to empirically establish clinically meaningful categories for the obesity awareness assessments. Fifth, as our study did not include a comparison group (i.e., lean ‘healthy’ control group or cross-condition comparison group), it remains unclear whether the parietal brain activation in relation to the fMRI obesity awareness task is unique to impaired obesity awareness versus a nonspecific finding common across multiple conditions that feature impaired illness awareness. Supporting the latter, the results of the current study (i.e., parietal brain activation in relation to impaired illness awareness) are similar to those found in other conditions using a similar fMRI illness awareness task (i.e., schizophrenia, substance use disorders (39, 51, 83)). Similar regional parietal brain activation across multiple clinical conditions in relation to illness awareness related processing supports the possible existence of a common, transdiagnostic subjective illness awareness/problem recognition-awareness-related brain network. Future fMRI studies using illness/problem awareness tasks could extend this work by including other clinical groups or ‘healthy’ participants.

Replication of the results in future studies will also clarify the role of the broader frontoparietal network in impaired obesity awareness and may help determine whether additional regions, beyond those identified here, are involved in impaired subjective obesity awareness.

Future research should also consider the shifting societal views toward obesity. The growing social acceptability and normalization of higher body weights, as promoted by today’s body-positivity and body acceptance movements, may inadvertently reduce individuals’ recognition of obesity and its associated health risks (23). As such, it would be beneficial for future studies to directly assess whether greater acceptance of obesity is associated with impaired obesity awareness, and how this relationship may impact clinical outcomes.

## 5 Conclusion

In conclusion, the results of this study, which revealed an association between impaired obesity awareness and increased BOLD signal in left PPA during an obesity awareness task, contribute to the limited literature on the neural correlates of impaired obesity awareness. The findings suggest that impaired illness awareness across different conditions may be related to similar frontoparietal network dysfunction. Given the current lack of established treatments for impaired obesity awareness, the frontoparietal network may be a potential therapeutic target to improve impaired obesity awareness, such as with non-invasive brain stimulation, which has shown promise in other conditions (75).

## Data Availability

The raw data supporting the conclusions of this article will be made available by the authors, without undue reservation.

## Footnotes

1
http://illnessawarenessscales.com/

## References

[1] World Health Organization. Obesity: preventing and managing the global epidemic World Health Organization (2000). 267 p. 11234459

[2] Frühbeck G Toplak H Woodward E Yumuk V Maislos M Oppert JM. Executive Committee of the European Association for the study of obesity obesity: the gateway to ill health - an EASO position statement on a rising public health, clinical and scientific challenge in Europe. Obes Facts. (2013) 6:117–20. doi: 10.1159/000350627, 23548858 PMC5644725

[3] Okunogbe A Nugent R Spencer G Powis J Ralston J Wilding J. Economic impacts of overweight and obesity: current and future estimates for 161 countries. BMJ glob. Health. (2022) 7:e009773. doi: 10.1136/bmjgh-2022-009773, 36130777 PMC9494015

[4] Okolo D Akpanumo B Okeke CH Aniekwe CE Ezenekwe EB Okobi OE . The influence of obesity on quality of life: a systematic review. J Adv Med Med Res. (2024) 36:267–79. doi: 10.9734/jammr/2024/v36i115637

[5] Inelmen EM Toffanello ED Enzi G Gasparini G Miotto F Sergi G . Predictors of drop-out in overweight and obese outpatients. Int J Obes. (2005) 29:122–8. doi: 10.1038/sj.ijo.0802846, 15545976

[6] Huisman S Maes S De Gucht VJ Chatrou M Haak HR. Low goal ownership predicts drop-out from a weight intervention study in overweight patients with type 2 diabetes. Int J Behav Med. (2010) 17:176–81. doi: 10.1007/s12529-009-9071-3, 20033629 PMC2910303

[7] Moroshko I Brennan L O’Brien P. Predictors of dropout in weight loss interventions: a systematic review of the literature. Obes Rev Off J Int Assoc Study Obes. (2011) 12:912–34. doi: 10.1111/j.1467-789X.2011.00915.x 21815990

[8] Zuckoff A. “Why won’t my patients do what’s good for them?” motivational interviewing and treatment adherence. Surg Obes Relat Dis Off J Am Soc Bariatr Surg. (2012) 8:514–21. doi: 10.1016/j.soard.2012.05.002 22704048

[9] Castellani W Ianni L Ricca V Mannucci E Rotella CM. Adherence to structured physical exercise in overweight and obese subjects: a review of psychological models. Eat Weight Disord EWD. (2003) 8:1–11. doi: 10.1007/BF03324983, 12762619

[10] Miller BML Brennan L. Measuring and reporting attrition from obesity treatment programs: a call to action! Obes Res Clin Pract. (2015) 9:187–202. doi: 10.1016/j.orcp.2014.08.007, 25293585

[11] Wing RR Phelan S. Long-term weight loss maintenance. Am J Clin Nutr. (2005) 82:222S–5S. https://linkinghub.elsevier.com/retrieve/pii/S0002916523295362 16002825 10.1093/ajcn/82.1.222S

[12] Fruh SM. Obesity: risk factors, complications, and strategies for sustainable long-term weight management. J Am Assoc Nurse Pract. (2017) 29:S3–S14. doi: 10.1002/2327-6924.12510 29024553 PMC6088226

[13] Smith NR Zivich PN Frerichs L. Social influences on obesity: current knowledge, emerging methods, and directions for future research and practice. Curr Nutr Rep. (2020) 9:31–41. doi: 10.1007/s13668-020-00302-8, 31960341 PMC7033640

[14] Robinson E. Overweight but unseen: a review of the underestimation of weight status and a visual normalization theory. Obes Rev. (2017) 18:1200–9. doi: 10.1111/obr.12570, 28730613 PMC5601193

[15] Ng M Dai X Cogen RM Abdelmasseh M Abdollahi A Abdullahi A . National-level and state-level prevalence of overweight and obesity among children, adolescents, and adults in the USA, 1990–2021, and forecasts up to 2050. Lancet. (2024) 404:2278–98. doi: 10.1016/S0140-6736(24)01548-4 39551059 PMC11694015

[16] Burke MA Heiland FW Nadler CM. From “overweight” to “about right”: evidence of a generational shift in body weight norms. Obes Silver Spring Md. (2010) 18:1226–34. doi: 10.1038/oby.2009.369 19875997

[17] Den Engelsen C Vos RC Rijken M Rutten GEHM. Comparison of perceptions of obesity among adults with central obesity with and without additional cardiometabolic risk factors and among those who were formally obese, 3 years after screening for central obesity. BMC Public Health. (2015) 15:1214. doi: 10.1186/s12889-015-2544-1 26644013 PMC4672514

[18] Johnson F Beeken RJ Croker H Wardle J. Do weight perceptions among obese adults in great britain match clinical definitions? Analysis of cross-sectional surveys from 2007 and 2012. BMJ Open. (2014) 4, 4:e005561. doi: 10.1136/bmjopen-2014-005561, 25394816 PMC4248082

[19] Johnson F Cooke L Croker H Wardle J. Changing perceptions of weight in Great Britain: comparison of two population surveys. BMJ. (2008) 337:a494–4. doi: 10.1136/bmj.a494, 18617488 PMC2500200

[20] Wetmore CM Mokdad AH. In denial: misperceptions of weight change among adults in the United States. Prev Med. (2012) 55:93–100. https://linkinghub.elsevier.com/retrieve/pii/S0091743512001624 22781370 10.1016/j.ypmed.2012.04.019

[21] Kilby R Mickelson KD. Combating weight-stigmatization in online spaces: the impacts of body neutral, body positive, and weight-stigmatizing TikTok content on body image and mood. Front Psych. (2025) 16. doi: 10.3389/fpsyt.2025.1577063/full PMC12185992 40557133

[22] Albert SL Massar RE Cassidy O Fennelly K Jay M Massey PM . Body positivity, physical health, and emotional well-being discourse on social media: content analysis of Lizzo’s Instagram. JMIR Form Res. (2024) 8:e60541. doi: 10.2196/60541 39496156 PMC11574494

[23] Muttarak R. Normalization of plus size and the danger of unseen overweight and obesity in England. Obes Silver Spring Md. (2018) 26:1125–9. doi: 10.1002/oby.22204 PMC6032838 29932517

[24] Babinski J. Contribution to the study of the mental disorders in hemiplegia of organic cerebral origin (anosognosia). Translated by K.G. Langer & D.N. Levine. Translated from the original Contribution à l’Étude des Troubles Mentaux dans l’Hémiplégie Organique Cérébrale (Anosognosie). Cortex; a journal devoted to the study of the nervous system and behavior. (1914) 61, 5–8. doi: 10.1016/j.cortex.2014.04.019. 25481462

[25] Ramachandran VS. Anosognosia in parietal lobe syndrome. Conscious Cogn. (1995) 4:22–51. https://linkinghub.elsevier.com/retrieve/pii/S1053810085710021 7497101 10.1006/ccog.1995.1002

[26] Amador XF. Awareness of illness in schizophrenia and schizoaffective and mood disorders. Arch Gen Psychiatry. (1994) 51:826–36. doi: 10.1001/archpsyc.1994.03950100074007, 7944872

[27] David A Buchanan A Reed A Almeida O. The assessment of insight in psychosis. Br J Psychiatry. (1992) 161:599–602. doi: 10.1192/bjp.161.5.599, 1422606

[28] Gerretsen P Remington G Borlido C Quilty L Hassan S Polsinelli G . The VAGUS insight into psychosis scale – self-report and clinician-rated versions. Psychiatry Res. (2014) 220:1084–9. doi: 10.1016/j.psychres.2014.08.005 25246410 PMC4470623

[29] Gerretsen P Kim J Shah P Quilty L Balakumar T Caravaggio F . OASIS: the obesity awareness and insight scale. Obes Med. (2018) 9:38–44. doi: 10.1016/j.obmed.2018.02.001, 30505975 PMC6260590

[30] Blokstra A Burns C Seidell J. Perception of weight status and dieting behaviour in dutch men and women. Int J Obes. (1999) 23:7–17. doi: 10.1038/sj.ijo.0800803, 10094580

[31] Bui AL Moscoso MG Bernabe-Ortiz A Checkley W Gilman RH Smeeth L . A secondary analysis examining the concordance of self-perception of weight and actual measurement of body fat percentage: the CRONICAS cohort study. BMC Obes. (2019) 6:9. doi: 10.1186/s40608-019-0229-5, 30984403 PMC6442421

[32] Duncan DT Wolin KY Scharoun-Lee M Ding EL Warner ET Bennett GG. Does perception equal reality? Weight misperception in relation to weight-related attitudes and behaviors among overweight and obese US adults. Int J Behav Nutr Phys Act. (2011) 8:20. doi: 10.1186/1479-5868-8-20, 21426567 PMC3073863

[33] Garay-Sevilla ME Malacara JM Gutiérrez-Roa A González E. Denial of disease in type 2 diabetes mellitus: its influence on metabolic control and associated factors. Diabet Med. (1999) 16:238–44. doi: 10.1046/j.1464-5491.1999.00033.x, 10227570

[34] Mogre V Nsoh JA Wanaba P Apala P. Demographic factors, weight management behaviours, receipt of healthcare professional’s counselling and having knowledge in basic anthropometric measurements associated with underassessment of weight status in overweight and obese type 2 diabetes patients. Obes Res Clin Pract. (2016) 10:381–9. doi: 10.1016/j.orcp.2015.08.018, 26385600

[35] Olsen SE Åsvold BO Frier BM Aune SE Hansen LI Bjørgaas MR. Hypoglycaemia symptoms and impaired awareness of hypoglycaemia in adults with type 1 diabetes: the association with diabetes duration. Diabet Med. (2014) 31:1210–7. doi: 10.1111/dme.12496 24824356

[36] Yaemsiri S Slining MM Agarwal SK. Perceived weight status, overweight diagnosis, and weight control among US adults: the NHANES 2003-2008 study. Int J Obes. (2011) 35:1063–70. doi: 10.1038/ijo.2010.229, 21042327

[37] Craig AD. How do you feel? Interoception: the sense of the physiological condition of the body. Nat Rev Neurosci. (2002) 3:655–66. doi: 10.1038/nrn894 12154366

[38] (Bud) Craig AD. How do you feel—now? The anterior insula and human awareness. Nat Rev Neurosci. (2009) 10:59–70. doi: 10.1038/nrn2555 19096369

[39] Gerretsen P Menon M Chakravarty MM Lerch JP Mamo DC Remington G . Illness denial in schizophrenia spectrum disorders: a function of left hemisphere dominance. Hum Brain Mapp. (2015) 36:213–25. doi: 10.1002/hbm.22624 25209949 PMC4268179

[40] Gerretsen P Menon M Mamo DC Fervaha G Remington G Pollock BG . Impaired insight into illness and cognitive insight in schizophrenia spectrum disorders: resting state functional connectivity. Schizophr Res. (2014) 160:43–50. doi: 10.1016/j.schres.2014.10.015 25458571 PMC4429527

[41] Orfei MD Robinson RG Prigatano GP Starkstein S Rusch N Bria P . Anosognosia for hemiplegia after stroke is a multifaceted phenomenon: a systematic review of the literature. Brain. (2007) 130:3075–90. doi: 10.1093/brain/awm106 17533170

[42] Orfei MD Robinson RG Bria P Caltagirone C Spalletta G. Unawareness of illness in neuropsychiatric disorders: phenomenological certainty versus etiopathogenic vagueness. Neuroscientist. (2008) 14:203–22. doi: 10.1177/1073858407309995 18057389

[43] Pia L. The anatomy of anosognosia for hemiplegia: a meta-analysis. Cortex. (2004) 40:367–77. doi: 10.1016/s0010-9452(08)70131-x 15156794

[44] Starkstein SE Jorge RE Robinson RG. The frequency, clinical correlates, and mechanism of anosognosia after stroke. Can J Psychiatr. (2010) 55:355–61. doi: 10.1177/070674371005500604 20540830

[45] Liemburg EJ van der Meer L Swart M Curcic-Blake B Bruggeman R Knegtering H . Reduced connectivity in the self-processing network of schizophrenia patients with poor insight. PLoS One. (2012) 7:e42707. doi: 10.1371/journal.pone.0042707, 22912723 PMC3415395

[46] Otte ML Schmitgen MM Wolf ND Kubera KM Calhoun VD Fritze S . Structure/function interrelationships and illness insight in patients with schizophrenia: a multimodal MRI data fusion study. Eur Arch Psychiatry Clin Neurosci. (2023) 273:1703–13. doi: 10.1007/s00406-023-01566-1, 36806586 PMC10713778

[47] Sapara A Ffytche DH Birchwood M Cooke MA Fannon D Williams SCR . Preservation and compensation: the functional neuroanatomy of insight and working memory in schizophrenia. Schizophr Res. (2014) 152:201–9. doi: 10.1016/j.schres.2013.11.026, 24332795 PMC3906535

[48] Van Der Meer L De Vos AE Stiekema APM Pijnenborg GHM Van Tol MJ Nolen WA . Insight in schizophrenia: involvement of self-reflection networks? Schizophr Bull. (2013) 39:1288–95. doi: 10.1093/schbul/sbs122, 23104865 PMC3796073

[49] Snelbaker AJ Wilkinson GS Robertson GJ Glutting JJ. (2001) Wide range achievement test 3 (WRAT3). In: Dorfman, W.I., Hersen, M. (eds). Understanding psychological assessment. Perspectives on Individual Differences. Boston, MA: Springer. doi: 10.1007/978-1-4615-1185-4_13 7080884

[50] Wilkinson GS. WRAT-3: wide range achievement test administration manual. Wilmington, Del: Wide Range, Inc. (1993).

[51] Kim J Plitman E Nakajima S Alshehri Y Iwata Y Chung JK . Modulation of brain activity with transcranial direct current stimulation: targeting regions implicated in impaired illness awareness in schizophrenia. Eur Psychiatry. (2019) 61:63–71. https://linkinghub.elsevier.com/retrieve/pii/S0924933819301051 31326732 10.1016/j.eurpsy.2019.06.007

[52] Menon M Schmitz TW Anderson AK Graff A Korostil M Mamo D . Exploring the neural correlates of delusions of reference. Biol Psychiatry. (2011) 70:1127–33. doi: 10.1016/j.biopsych.2011.05.037, 21831358

[53] Sheehan DV Lecrubier Y Sheehan KH Amorim P Janavs J Weiller E . The mini-international neuropsychiatric interview (M.I.N.I.): the development and validation of a structured diagnostic psychiatric interview for DSM-IV and ICD-10. J Clin Psychiatry. (1998) 59:22–33. quiz 34–57 9881538

[54] Broadbent E Petrie KJ Main J Weinman J. The brief illness perception questionnaire. J Psychosom Res. (2006) https://linkinghub.elsevier.com/retrieve/pii/S0022399905004915) 60:631–7. 16731240 10.1016/j.jpsychores.2005.10.020

[55] Beck AT Steer RA Brown GK. Beck depression inventory. (1996).

[56] Spitzer RL Kroenke K Williams JBW Löwe B. A brief measure for assessing generalized anxiety disorder: the GAD-7. Arch Intern Med. (2006) 166:1092. doi: 10.1001/archinte.166.10.1092 16717171

[57] Gormally J Black S Daston S Rardin D. The assessment of binge eating severity among obese persons. Addict Behav. (1982) 7:47–55. doi: 10.1016/0306-4603(82)90024-7 7080884

[58] Ashburner J Friston KJ. Unified segmentation. NeuroImage. (2005) 26:839–51. https://linkinghub.elsevier.com/retrieve/pii/S1053811905001102 15955494 10.1016/j.neuroimage.2005.02.018

[59] Gerretsen P Chakravarty MM Mamo D Menon M Pollock BG Rajji TK . Frontotemporoparietal asymmetry and lack of illness awareness in schizophrenia. Hum Brain Mapp. (2013) 34:1035–43. doi: 10.1002/hbm.21490, 22213454 PMC6870294

[60] Carter CS Heckers S Nichols T Pine DS Strother S. Optimizing the design and analysis of clinical functional magnetic resonance imaging research studies. Biol Psychiatry. (2008) 64:842–9. doi: 10.1016/j.biopsych.2008.06.014 18718572

[61] Rolls ET Joliot M Tzourio-Mazoyer N. Implementation of a new parcellation of the orbitofrontal cortex in the automated anatomical labeling atlas. NeuroImage. (2015) 122:1–5. doi: 10.1016/j.neuroimage.2015.07.075 26241684

[62] Johnson F Beeken RJ Croker H Wardle J. Do weight perceptions among obese adults in Great Britain match clinical definitions? Analysis of cross-sectional surveys from 2007 and 2012. BMJ Open. (2014) 4:e005561. doi: 10.1136/bmjopen-2014-005561 PMC4248082 25394816

[63] Johnson F Cooke L Croker H Wardle J. Changing perceptions of weight in Great Britain: comparison of two population surveys. BMJ. (2008) 337:a494. doi: 10.1136/bmj.a494 18617488 PMC2500200

[64] Mizrahi T Axelrod V. Similarity in activity and laterality patterns in the angular gyrus during autobiographical memory retrieval and self-referential processing. Brain Struct Funct. (2023) 228:219–38. doi: 10.1007/s00429-022-02569-9, 36166073

[65] Mohan A Roberto AJ Mohan A Lorenzo A Jones K Carney MJ . The significance of the default mode network (DMN) in neurological and neuropsychiatric disorders: a review. Yale J Biol Med. (2016) 89:49–57. 27505016 PMC4797836

[66] Herzog N Hartmann H Janssen LK Waltmann M Fallon SJ Deserno L . Working memory gating in obesity: insights from a case-control fMRI study. Appetite. (2024) 195:107179. doi: 10.1016/j.appet.2023.107179 38145879

[67] Smith R Lane RD Alkozei A Bao J Smith C Sanova A . The role of medial prefrontal cortex in the working memory maintenance of one’s own emotional responses. Sci Rep. (2018) 8:3460. doi: 10.1038/s41598-018-21896-8 PMC5823866 29472625

[68] Hartmann H Janssen LK Herzog N Morys F Fängström D Fallon SJ . Self-reported intake of high-fat and high-sugar diet is not associated with cognitive stability and flexibility in healthy men. Appetite. (2023) 183:106477. doi: 10.1016/j.appet.2023.106477 36764221

[69] Shad MU Tamminga CA Cullum M Haas GL Keshavan MS. Insight and frontal cortical function in schizophrenia: a review. Schizophr Res. (2006) 86:54–70. doi: 10.1016/j.schres.2006.06.006, 16837168

[70] Soldevila-Matías P Schoretsanitis G Tordesillas-Gutierrez D Cuesta MJ De Filippis R Ayesa-Arriola R . Neuroimaging correlates of insight in non-affective psychosis: a systematic review and meta-analysis. Rev Psiquiatr Salud Ment Engl Ed. (2022) 15:117–33. doi: 10.1016/j.rpsm.2021.07.001 35840278

[71] Berti A Bottini G Gandola M Pia L Smania N Stracciari A . Shared cortical anatomy for motor awareness and motor control. Science. (2005) 309:488–91. doi: 10.1126/science.1110625, 16020740

[72] Karnath HO. Awareness of the functioning of one’s own limbs mediated by the insular cortex? J Neurosci. (2005) 25:7134–8. doi: 10.1523/JNEUROSCI.1590-05.2005, 16079395 PMC6725240

[73] Ćurčić-Blake B Van Der Meer L Pijnenborg GHM David AS Aleman A. Insight and psychosis: functional and anatomical brain connectivity and self-reflection in S chizophrenia. Hum Brain Mapp. (2015) 36:4859–68. doi: 10.1002/hbm.22955, 26467308 PMC6869637

[74] Xavier RM Vorderstrasse A. Neurobiological basis of insight in schizophrenia: a systematic review. Nurs Res. (2016) 65:224–37. doi: 10.1097/NNR.0000000000000159 27124258

[75] Adam O Blay M Brunoni AR Chang HA Gomes JS Javitt DC . Efficacy of transcranial direct current stimulation to improve insight in patients with schizophrenia: a systematic review and meta-analysis of randomized controlled trials. Schizophr Bull. (2022) 48:1284–94. doi: 10.1093/schbul/sbac078 35820035 PMC9673267

[76] Gerretsen P Diaz P Mamo D Kavanagh D Menon M Pollock BG . Transient insight induction with electroconvulsive therapy in a patient with refractory schizophrenia: a case report and systematic literature review. J ECT. (2011) 27:247–50. doi: 10.1097/YCT.0b013e3181f816f6, 20966768

[77] Haesebaert F Mondino M Saoud M Poulet E Brunelin J. Efficacy and safety of fronto-temporal transcranial random noise stimulation (tRNS) in drug-free patients with schizophrenia: a case study. Schizophr Res. (2014) 159:251–2. doi: 10.1016/j.schres.2014.07.043, 25129852

[78] Kallel L Mondino M Brunelin J. Effects of theta-rhythm transcranial alternating current stimulation (4.5 hz-tACS) in patients with clozapine-resistant negative symptoms of schizophrenia: a case series. J Neural Transm. (2016) 123:1213–7. doi: 10.1007/s00702-016-1574-x 27194229

[79] Reddan MC Lindquist MA Wager TD. Effect size estimation in neuroimaging. JAMA. Psychiatry. (2017) 74:207–8. doi: 10.1001/jamapsychiatry.2016.3356, 28099973

[80] Mogre V Aleyira S Nyaba R. Misperception of weight status and associated factors among undergraduate students. Obes Res Clin Pract. (2015) 9:466–74. https://linkinghub.elsevier.com/retrieve/pii/S1871403X15000344 25842980 10.1016/j.orcp.2015.03.002

[81] Leenaerts N Jongen D Ceccarini J Van Oudenhove L Vrieze E. The neurobiological reward system and binge eating: a critical systematic review of neuroimaging studies. Int J Eat Disord. (2022) 55:1421–58. doi: 10.1002/eat.23776 35841198

[82] Waltmann M Herzog N Reiter AMF Villringer A Horstmann A Deserno L. Neurocomputational mechanisms underlying differential reinforcement learning from wins and losses in obesity with and without binge eating. Biol Psychiatry Cogn Neurosci Neuroimaging. (2024) 9:1281–90. doi: 10.1016/j.bpsc.2024.06.002 38909896

[83] Kyte S Song J Nikolova YS Ruocco AC Agarwal SM Amaev A . The neural correlates of illness awareness in addiction: a pilot exploratory analysis of preliminary data from the cognitive dysfunction in the addictions (CDiA). research program Front Neurol. (2026):1694826:16. doi: 10.3389/fneur.2025.1694826/full 41561341 PMC12812657

[84] Lancaster JL Summerlin JL Rainey L Freitas CS Fox PT. The talairach daemon a database server for talairach atlas labels. NeuroImage. (1997) 5 (4 PART II), S633.

[85] Lancaster JL Woldorff MG Parsons LM Liotti M Freitas CS Rainey L . Automated talairach atlas labels for functional brain mapping. Hum Brain Mapp. (2000) 10:120–31. doi: 10.1002/1097-0193(200007)10:3<120::AID-HBM30>3.0.CO;2-8, 10912591 PMC6871915

[86] Maldjian JA Laurienti PJ Kraft RA Burdette JH. An automated method for neuroanatomic and cytoarchitectonic atlas-based interrogation of fMRI data sets. NeuroImage. (2003) 19:1233–9. doi: 10.1016/S1053-8119(03)00169-1, 12880848

