# Supplementary material for: Neural correlates of illness awareness in obesity: an exploratory pilot fMRI study
Source: Front Neurol. 2026 Mar 23;17:1675116. doi: 10.3389/fneur.2026.1675116 (PMC13051503; doi:10.3389/fneur.2026.1675116)
Supplement: Supplementary file 1 [file Data_Sheet_1.docx]

| **Supplemental Table 1. Regional activations for the second-level contrast – regression** | | | | | | | | | |
| --- | --- | --- | --- | --- | --- | --- | --- | --- | --- |
|  | **Adjusted for age, gender** | | | | | | | | |
|  | Cluster Maxima | | | Cluster size | t value | *p* value uncorrected | *p* value FWE corrected | AAL Mask for correction | *p* value FWE small volume corrected |
|  | x | y | z |  |  |  |  |  |  |
| **Illness-related > Control stimuli** | | | | | | | | | |
| **Impaired** | | | | | | | | | |
| Right parahippocampal gyrus | 27 | -25 | -16 | 33 | 5.02 | <0.001 | 0.297 | - | - |
| Right cerebellum crus I | 45 | -52 | -34 | 14 | 4.98 | <0.001 | 0.317 | - | - |
| Left angular gyrus | -42 | -58 | 23 | 48 | 4.81 | <0.001 | 0.405 | Left angular gyrus | 0.007* |
| Left fusiform gyrus | -48 | -58 | -16 | 11 | 4.30 | <0.001 | 0.737 | - | - |
| Right angular gyrus | 54 | -64 | 23 | 13 | 4.29 | <0.001 | 0.742 | Right angular gyrus | 0.027* |
| Left superior temporal gyrus | -51 | -7 | -7 | 2 | 4.22 | <0.001 | 0.781 | - | - |
| Cerebellar vermis | 0 | -34 | -7 | 20 | 4.13 | <0.001 | 0.832 | - | - |
| Left thalamus | -3 | -7 | 5 | 11 | 4.04 | <0.001 | 0.878 | - | - |
| Left fusiform gyrus | -39 | -40 | -19 | 23 | 3.99 | <0.001 | 0.899 | - | - |
| Left amygdala | -39 | -1 | -19 | 13 | 3.94 | <0.001 | 0.917 | - | - |
| Ventricle | 3 | 20 | 8 | 7 | 3.94 | <0.001 | 0.918 | - | - |
| Left superior occipital gyrus | -21 | -64 | 29 | 6 | 3.92 | <0.001 | 0.924 | - | - |
| Right supramarginal gyrus | 54 | -37 | 23 | 3 | 3.89 | <0.001 | 0.933 | Right supramarginal gyrus | 0.063 |
| Left amygdala | -21 | -1 | -13 | 2 | 3.85 | <0.001 | 0.945 | - | - |
| Left posterior cingulate gyrus | -12 | -46 | 29 | 7 | 3.84 | <0.001 | 0.948 | - | - |
| Right inferior temporal gyrus | 45 | -37 | -13 | 2 | 3.75 | 0.001 | 0.969 | - | - |
| Right inferior temporal gyrus | 54 | -43 | -16 | 2 | 3.74 | 0.001 | 0.971 | - | - |
| Right cerebellum | 33 | -46 | -40 | 1 | 3.68 | 0.001 | 0.979 | - | - |
| Left parahippocampal gyrus | -18 | -34 | -10 | 2 | 3.65 | 0.001 | 0.983 | - | - |
| Right middle temporal gyrus | 54 | -58 | 8 | 1 | 3.59 | 0.001 | 0.989 | - | - |
| right superior temporal gyrus | 60 | -16 | 8 | 1 | 3.58 | 0.001 | 0.990 | - | - |
| Right middle temporal gyrus | 63 | -49 | 17 | 2 | 3.56 | 0.001 | 0.991 | - | - |
| Right middle temporal gyrus | 60 | -1 | -16 | 2 | 3.54 | 0.001 | 0.992 | - | - |
| Right cerebellum | 33 | -46 | -28 | 1 | 3.54 | 0.001 | 0.993 | - | - |
| Right fusiform gyrus | 36 | -55 | -7 | 2 | 3.53 | 0.001 | 0.993 | - | - |
| Left cerebellum | -21 | -67 | -46 | 1 | 3.53 | 0.001 | 0.993 | - | - |
| **Intact** |  |  |  |  |  |  |  | | |
| Nil |  |  |  |  |  |  |  | | |
|  | | | | | | | | | |
| **General Illness awareness > Control stimuli** | | | | | | | | | |
| **Impaired** | | | | | | | | | |
| Left angular gyrus | -39 | -58 | 23 | 91 | 6.84 | <0.001 | 0.011* | Left angular gyrus | <0.001* |
| Left fusiform gyrus | -39 | -43 | -19 | 25 | 4.90 | <0.001 | 0.340 | - | - |
| Left calcarine fissure and  surrounding cortex | -15 | -49 | 5 | 9 | 4.53 | <0.001 | 0.558 | - | - |
| Right angular gyrus | 39 | -67 | 44 | 12 | 4.20 | <0.001 | 0.778 | Right angular gyrus | 0.031* |
| Left inferior frontal gyrus, opercular part | -33 | 17 | 26 | 11 | 4.14 | <0.001 | 0.811 | Left middle frontal region | 0.124 |
| Left precentral gyrus | -45 | -1 | 53 | 6 | 4.07 | <0.001 | 0.848 | - | - |
| Left parahippocampal gyrus | -18 | -34 | -7 | 4 | 4.06 | <0.001 | 0.854 | - | - |
| Left superior occipital lobe | -21 | -64 | 29 | 5 | 3.96 | <0.001 | 0.896 | - | - |
| Right supramarginal gyrus | 66 | -22 | 14 | 3 | 3.95 | <0.001 | 0.902 | Right supramarginal gyrus | 0.098 |
| Right cerebellum crus I | 42 | -49 | -34 | 2 | 3.84 | 0.001 | 0.909 | - | - |
| Right middle temporal gyrus | 54 | -64 | 23 | 3 | 3.88 | <0.001 | 0.928 | - | - |
| Left thalamus | -6 | -7 | 5 | 2 | 3.85 | <0.001 | 0.937 | - | - |
| Cerebellar vermis | 6 | -34 | -7 | 4 | 3.78 | 0.001 | 0.954 | - | - |
| Right superior frontal gyrus | 21 | 32 | 53 | 1 | 3.76 | 0.001 | 0.960 | Right superior frontal region | 0.159 |
| Right parahippocampal gyrus | 27 | -25 | -16 | 2 | 3.73 | 0.001 | 0.966 | - | - |
| Right insula | 39 | -22 | 2 | 1 | 3.59 | 0.001 | 0.985 | Right insula | 0.108 |
| Right caudate | 6 | 17 | 11 | 2 | 3.59 | 0.001 | 0.986 | - | - |
| Left superior frontal gyrus | -12 | 17 | 56 | 1 | 3.57 | 0.001 | 0.988 | Left superior frontal region | 0.206 |
| Left caudate nucleus | -15 | 5 | 17 | 1 | 3.57 | 0.001 | 0.988 | - | - |
| Right cerebellum | 33 | -46 | -43 | 1 | 3.53 | 0.001 | 0.991 | - | - |
| Left middle frontal gyrus | -48 | 17 | 41 | 1 | 3.53 | 0.001 | 0.991 | - | - |
| **Intact** |  |  |  |  |  |  |  |  |  |
| Nil |  |  |  |  |  |  |  |  |  |
|  | | | | | | | |  |  |
| **Symptom Awareness > Control stimuli** | | | | | | | | | |
| **Impaired** | | | | | | | | | |
| Right cerebellum crus I | 45 | -52 | -34 | 9 | 4.85 | <0.001 | 0.398 | - | - |
| Left inferior occipital gyrus | -51 | -58 | -13 | 31 | 4.65 | <0.001 | 0.520 | - | - |
| Left middle temporal/Left angular gyrus | -30 | -58 | 20 | 51 | 4.53 | <0.001 | 0.599 | Left angular gyrus | 0.023* |
| Right superior temporal gyrus | 60 | -16 | 8 | 2 | 4.44 | <0.001 | 0.656 | - | - |
| Right inferior temporal gyrus | 39 | -55 | -7 | 9 | 4.41 | <0.001 | 0.680 | - | - |
| Right cerebellum crus I | 30 | -55 | -40 | 11 | 4.23 | <0.001 | 0.794 | - | - |
| Right inferior temporal gyrus | 54 | -46 | -13 | 7 | 4.20 | <0.001 | 0.806 | - | - |
| Ventricle | 27 | -43 | 14 | 18 | 4.02 | <0.001 | 0.898 | - | - |
| Right angular gyrus | 54 | -64 | 23 | 5 | 4.01 | <0.001 | 0.898 | Right angular gyrus | 0.048* |
| Left inferior parietal gyrus | -45 | -25 | 41 | 9 | 3.91 | <0.001 | 0.936 | Left inferior parietal region | 0.105 |
| Left superior temporal gyrus | -51 | -4 | -10 | 3 | 3.87 | <0.001 | 0.947 | - | - |
| Left Heschl gyrus | -27 | -28 | 20 | 6 | 3.87 | <0.001 | 0.948 | - | - |
| Ventricle | 0 | 20 | 8 | 5 | 3.81 | <0.001 | 0.962 | - | - |
| Left middle temporal gyrus | -45 | -1 | -22 | 1 | 3.78 | 0.001 | 0.968 | - | - |
| Cerebellar vermis | -3 | -34 | -4 | 5 | 3.77 | 0.001 | 0.968 | - | - |
| Right parahippocampal | 27 | -25 | -13 | 3 | 3.72 | 0.001 | 0.977 | - | - |
| Left cerebellum crus II | -21 | -67 | -46 | 2 | 3.71 | 0.001 | 0.979 | - | - |
| Left inferior occipital | -30 | -79 | -1 | 4 | 3.66 | 0.001 | 0.985 | - | - |
| Right amygdala | 33 | 2 | -22 | 4 | 3.64 | 0.001 | 0.986 | - | - |
| Cerebellar vermis | 6 | -37 | -16 | 1 | 3.66 | 0.001 | 0.988 | - | - |
| Left amygdala | -39 | -1 | -19 | 1 | 3.61 | 0.001 | 0.989 | - | - |
| Right inferior frontal gyrus, triangular part | 36 | 23 | 11 | 2 | 3.58 | 0.001 | 0.992 | Right insula | 0.121 |
| **Intact** |  |  |  |  |  |  |  |  |  |
| Nil |  |  |  |  |  |  |  |  |  |
|  | | | | | | | | | |
| **Need for Treatment > Control stimuli** | | | | | | | | | |
| **Impaired** | | | | | | | | | |
| Cerebellar vermis | 6 | -37 | -13 | 87 | 5.86 | <0.001 | 0.069 | - | - |
| Right precuneus | 21 | -40 | 5 | 25 | 5.65 | <0.001 | 0.101 | - | - |
| Left angular gyrus | -39 | -58 | 23 | 20 | 4.83 | <0.001 | 0.383 | Left angular gyrus | 0.006* |
| Left inferior occipital gyrus | -51 | -58 | -13 | 8 | 4.44 | <0.001 | 0.628 | - | - |
| Right parahippocampal gyrus | 24 | -25 | -16 | 8 | 4.37 | <0.001 | 0.677 | - | - |
| Left superior temporal gyrus | -51 | -7 | -7 | 2 | 4.25 | <0.001 | 0.754 | - | - |
| Right caudate nucleus | 0 | 20 | 8 | 10 | 4.13 | <0.001 | 0.825 | - | - |
| Right angular gyrus | 51 | -61 | 20 | 10 | 4.01 | <0.001 | 0.882 | Right angular gyrus | 0.077 |
| Right middle temporal gyrus | 45 | -37 | -13 | 3 | 3.91 | <0.001 | 0.920 | - | - |
| Left inferior temporal gyrus | -30 | -37 | -19 | 3 | 3.91 | <0.001 | 0.922 | - | - |
| Left superior temporal pole | -27 | 5 | -19 | 3 | 3.83 | <0.001 | 0.946 | - | - |
| Left fusiform gyrus | -39 | -1 | -19 | 3 | 3.82 | <0.001 | 0.948 | - | - |
| Right cerebellum crus I | 45 | -52 | -34 | 3 | 3.82 | <0.001 | 0.950 | - | - |
| Cerebelum | 30 | -40 | -31 | 1 | 3.60 | 0.001 | 0.986 | - | - |
| **Intact** |  |  |  |  |  |  |  |  |  |
| Nil |  |  |  |  |  |  |  |  |  |
| Threshold: p<0.001, uncorr., 0 voxels.  *p<0.05 | | | | | | | | | |

| **Table 3. Regional activations for the second-level contrast – group comparison** | | | | | | | | | | |
| --- | --- | --- | --- | --- | --- | --- | --- | --- | --- | --- |
| **Adjusted for age, gender; threshold: p<0.001, uncorr.,0 voxels** | | | | | | | | | | |
|  | Cluster Maxima | | | | Cluster size | t value | *p* value uncorrected | *p* value FWE corrected |  | *p* value FWE small volume corrected |
|  | x | | y | z |  |  |  |  |  |  |
| **Illness-related > Control stimuli**  **Impaired > Intact** | | | | | | | | | | |
| Right hippocampus | 27 | | -34 | -4 | 90 | 5.22 | <0.001 | 0.220 |  | - |
| Left amygdala | -30 | | 2 | -19 | 20 | 5.13 | <0.001 | 0.256 |  | - |
| Left superior temporal gyrus | -51 | | -7 | -10 | 17 | 4.86 | <0.001 | 0.385 |  | - |
| Left thalamus | -6 | | -7 | 5 | 13 | 4.63 | <0.001 | 0.526 |  | - |
| Left cerebellum | -36 | | -49 | -25 | 36 | 4.49 | <0.001 | 0.616 |  | - |
| Right cerebellum crus I | 45 | | -52 | -34 | 8 | 4.48 | <0.001 | 0.621 |  | - |
| Right middle temporal gyrus | 54 | | -61 | 14 | 34 | 4.40 | <0.001 | 0.674 |  | - |
| Left angular gyrus | -45 | | -61 | 23 | 28 | 4.37 | <0.001 | 0.697 |  | 0.017* |
| Left fusiform gyrus | -27 | | -37 | -19 | 42 | 4.35 | <0.001 | 0.710 |  | - |
| Right superior frontal gyrus | 21 | | 32 | 53 | 3 | 4.21 | <0.001 | 0.795 |  | - |
| Left cerebellum | -9 | | -52 | -46 | 8 | 4.21 | <0.001 | 0.797 |  | - |
| Left superior occipital gyrus | -21 | | -64 | 29 | 9 | 4.13 | <0.001 | 0.841 |  | - |
| Left fusiform gyrus | -12 | | -46 | 8 | 7 | 4.03 | <0.001 | 0.885 |  | - |
| Left inferior temporal gyrus | -48 | | -55 | -16 | 12 | 4.03 | <0.001 | 0.887 |  | - |
| Right inferior occipital gyrus | 45 | | -61 | -13 | 12 | 3.98 | <0.001 | 0.909 |  | - |
| Right superior temporal gyrus | 60 | | -37 | 20 | 7 | 3.94 | <0.001 | 0.922 |  | 0.078 |
| Left inferior parietal gyrus | -45 | | -25 | 44 | 4 | 3.82 | <0.001 | 0.955 |  | - |
| Left lingual | -3 | | -58 | 8 | 3 | 3.72 | 0.001 | 0.975 |  | - |
| Cerebellar vermis | 6 | | -37 | -16 | 1 | 3.66 | 0.001 | 0.983 |  | - |
| Right calcarine fissure and  surrounding cortex | 6 | | -55 | 14 | 1 | 3.62 | 0.001 | 0.988 |  | - |
| Left middle temporal gyrus | -51 | | -49 | 20 | 1 | 3.60 | 0.001 | 0.989 |  | - |
| Left inferior frontal gyrus, (opercular part) | 36 | | 23 | -13 | 1 | 3.57 | 0.001 | 0.991 |  | - |
| Left inferior parietal gyrus | -36 | | -46 | 50 | 1 | 3.53 | 0.001 | 0.994 |  | 0.148 |
| Right superior parietal lobule | 30 | | -58 | -40 | 1 | 3.51 | 0.001 | 0.995 |  | - |
|  | | **Intact > Impaired** | | | | | | | | |
| Nil |  | |  |  |  |  |  |  |  |  |
|  | | | | | | | | |  |  |
| **General Illness awareness > Neutral** | | | | | | | | |  |  |
| **Impaired > Intact** | | | | | | | | |  |  |
| Left angular gyrus | -42 | | -58 | 20 | 30 | 5.03 | <0.001 | 0.298 |  | 0.006* |
| Right superior frontal gyrus | 21 | | 32 | 53 | 4 | 4.27 | <0.001 | 0.761 |  | - |
| Left cerebellum | -12 | | -52 | -46 | 9 | 4.20 | <0.001 | 0.802 |  | - |
| Left amygdala | -30 | | 2 | -16 | 5 | 4.17 | <0.001 | 0.817 |  | - |
| Left hippocampus | -21 | | -31 | -4 | 5 | 4.13 | <0.001 | 0.841 |  | - |
| Left precuneus | -3 | | -55 | 14 | 15 | 4.13 | <0.001 | 0.857 |  | - |
| Right angular gyrus | 39 | | -70 | 44 | 2 | 4.06 | <0.001 | 0.876 |  | 0.044* |
| Left thalamus | -6 | | -7 | 5 | 6 | 4.06 | <0.001 | 0.876 |  | - |
| Left cerebellum | -36 | | -46 | -25 | 6 | 3.98 | <0.001 | 0.906 |  | - |
| Left fusiform gyrus | -24 | | -37 | -19 | 10 | 3.87 | <0.001 | 0.945 |  | - |
| Left thalamus | -9 | | -28 | -1 | 4 | 3.85 | <0.001 | 0.948 |  | - |
| Left precentral gyrus | -39 | | 4 | 50 | 4 | 3.84 | <0.001 | 0.951 |  | - |
| Right precuneus | 18 | | -43 | 2 | 3 | 3.81 | <0.001 | 0.958 |  | - |
| Right hippocampus | 24 | | -34 | -4 | 3 | 3.81 | <0.001 | 0.959 |  | - |
| Left middle temporal gyrus | -54 | | -4 | -16 | 8 | 3.74 | 0.001 | 0.972 |  | - |
| Left calcarine fissure and  surrounding cortex | -12 | | -46 | 8 | 1 | 3.69 | 0.001 | 0.979 |  | - |
|  | | **Intact > Impaired** | | | | | | | | |
| Nil |  | |  |  |  |  |  |  |  |  |
|  | | | | | | | | |  |  |
| **Symptom Awareness > Neutral** | | | | | | | | |  |  |
| **Impaired > Intact** | | | | | | | | |  |  |
| Left superior temporal gyrus | -51 | | -4 | -10 | 21 | 5.63 | <0.001 | 0.114 |  | v |
| Left amygdala | -33 | | -1 | -19 | 22 | 5.56 | <0.001 | 0.127 |  | - |
| Left cerebellum | -36 | | -58 | -25 | 58 | 4.80 | <0.001 | 0.434 |  | - |
| Right parahippocampal gyrus | 27 | | -25 | -13 | 35 | 4.52 | <0.001 | 0.611 |  | - |
| Right middle temporal gyrus | 54 | | -61 | 14 | 33 | 4.47 | <0.001 | 0.647 |  | - |
| Right inferior temporal gyrus | 39 | | -58 | -7 | 30 | 4.39 | <0.001 | 0.694 |  | - |
| Left parahippocampal gyrus | -18 | | -34 | -13 | 5 | 4.39 | <0.001 | 0.697 |  | - |
| Right cerebellum crus I | 45 | | -52 | -34 | 8 | 4.11 | <0.001 | 0.863 |  | - |
| Left inferior parietal gyrus | -45 | | -25 | 41 | 5 | 4.08 | <0.001 | 0.876 |  | - |
| Right cerebellum | 30 | | -58 | -40 | 6 | 3.98 | <0.001 | 0.916 |  | - |
| Left cerebellum | -33 | | -61 | -49 | 5 | 3.91 | <0.001 | 0.937 |  | - |
| Left angular gyrus | -48 | | -61 | 23 | 4 | 3.88 | <0.001 | 0.948 |  | 0.045* |
| Left precuneus | -6 | | -61 | 17 | 4 | 3.76 | 0.001 | 0.972 |  | - |
| Left superior parietal lobule | -27 | | -52 | 62 | 2 | 3.72 | 0.001 | 0.979 |  | 0.096 |
| Left superior occipital gyrus | -24 | | -64 | 26 | 2 | 3.71 | 0.001 | 0.980 |  | - |
| Left fusiform gyrus | -27 | | -37 | -19 | 6 | 3.67 | 0.001 | 0.985 |  | - |
| Right superior temporal pole | 33 | | 5 | -22 | 1 | 3.65 | 0.001 | 0.987 |  | - |
| Right inferior frontal gyrus, (triangular part) | 36 | | 23 | 11 | 1 | 3.62 | 0.001 | 0.989 |  | - |
| Left middle temporal gyrus | -51 | | -67 | 20 | 2 | 3.57 | 0.001 | 0.993 |  | - |
| Right supramarginal gyrus | 60 | | -40 | 23 | 3 | 3.54 | 0.001 | 0.995 |  | 0.126 |
| Right inferior temporal gyrus | 57 | | -49 | -10 | 1 | 3.52 | 0.001 | 0.995 |  | - |
| Right middle occipital gyrus | 36 | | -76 | 32 | 1 | 3.52 | 0.001 | 0.995 |  | - |
| **Intact > Impaired** | | | | | | | | | | |
| Nil |  | |  |  |  |  |  |  |  |  |
|  | | | | | | | | |  |  |
| **Need for Treatment > Neutral** | | | | | | | | |  |  |
| **Impaired > Intact** | | | | | | | | |  |  |
| Right precuneus | 21 | | -40 | 2 | 73 | 5.19 | <0.001 | 0.232 |  | - |
| Left fusiform gyrus | -15 | | -37 | -10 | 40 | 4.84 | <0.001 | 0.396 |  | - |
| Right inferior frontal gyrus, (orbital part) | 36 | | 23 | -13 | 19 | 4.70 | <0.001 | 0.477 |  | 0.078 |
| Left superior temporal gyrus | -51 | | -4 | -10 | 6 | 4.52 | <0.001 | 0.594 |  | - |
| Left superior temporal pole | -36 | | 20 | -22 | 13 | 4.49 | <0.001 | 0.617 |  | - |
| Right middle temporal gyrus | 54 | | -61 | 17 | 25 | 4.45 | <0.001 | 0.642 |  | - |
| Left middle temporal/Left angular gyrus | -42 | | -58 | 20 | 11 | 4.33 | <0.001 | 0.718 |  | 0.045* |
| Left inferior frontal gyrus, (triangular part) | -54 | | 23 | 5 | 15 | 4.33 | <0.001 | 0.719 |  | - |
| Left inferior occipital gyrus | -51 | | -58 | -13 | 9 | 4.23 | <0.001 | 0.781 |  | - |
| Left cerebellum crus I | -36 | | -61 | -28 | 8 | 4.07 | <0.001 | 0.867 |  | - |
| Left superior temporal pole | -27 | | 5 | -19 | 8 | 4.03 | <0.001 | 0.884 |  | - |
| Right cerebellum crus I | 45 | | -52 | -34 | 3 | 3.99 | <0.001 | 0.901 |  | - |
| Cerebellar vermis | 6 | | -37 | -16 | 2 | 3.81 | <0.001 | 0.957 |  | - |
| Right superior temporal pole | 33 | | 5 | -22 | 2 | 3.77 | 0.001 | 0.966 |  | - |
| Left cerebellum crus II | -39 | | -64 | -46 | 1 | 3.75 | 0.001 | 0.970 |  | - |
| Right superior parietal lobule | 18 | | -61 | 62 | 1 | 3.72 | 0.001 | 0.975 |  | 0.098 |
| Left superior occipital gyrus | -21 | | -64 | 29 | 3 | 3.60 | 0.001 | 0.989 |  | - |
| Cerebellar vermis | -6 | | -34 | -4 | 2 | 3.57 | 0.001 | 0.991 |  | - |
| Right cerebellum | 27 | | -40 | -31 | 2 | 3.57 | 0.001 | 0.991 |  | - |
|  | | **Intact > Impaired** | | | | | | | | |
| Nil |  | |  |  |  |  |  |  |  |  |
| Threshold: p<0.001, uncorr., 0 voxels  *p<0.05 | | | | | | | | |  |  |
